# Supplementary material for: Mechanical loading and hyperosmolarity as a daily resetting cue for skeletal circadian clocks
Source: Nat Commun. 2023 Nov 14;14:7237. doi: 10.1038/s41467-023-42056-1 (PMC10646113; doi:10.1038/s41467-023-42056-1)
Supplement: Supplementary file 1 — Supplementary Information [file 41467_2023_42056_MOESM1_ESM.pdf]

Supplementary Figures and legends:

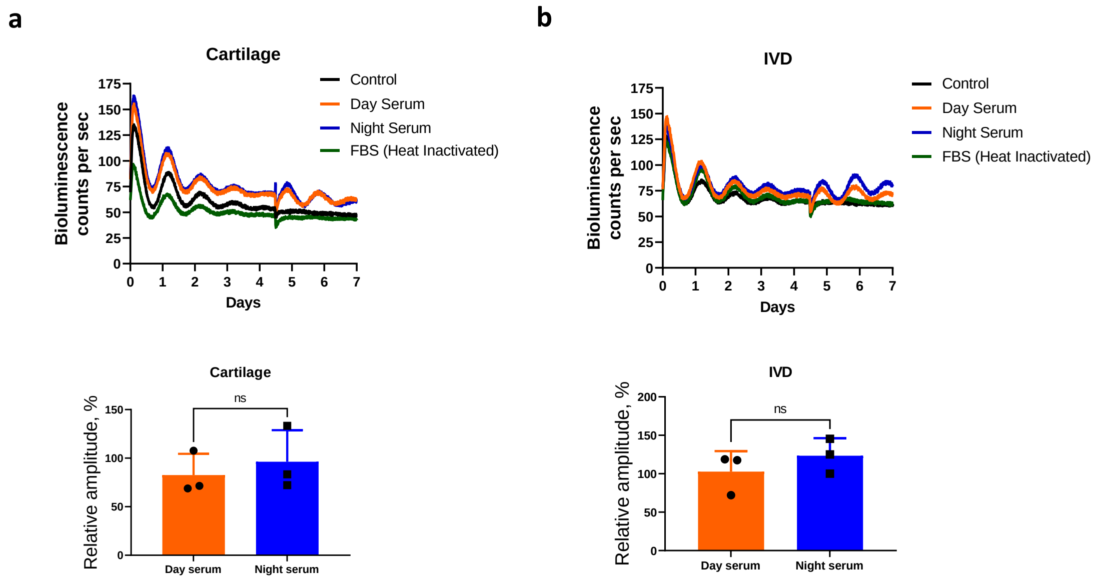

Figure S1. Comparison of synchronising effects of day and night serum on cartilage and IVD explants. Bioluminescence recording of PER2::Luc mouse femoral head cartilage (**a**) and IVD (**b**) explants. At day 4 of the explant cultures 10% mouse serum harvested during day (orange), during night (blue), 10% heat inactivated FBS (green) or 10% volume fresh medium (black) were added. Amplitude of the peak after treatment was quantified as % of the peak at day 1 of recording. Statistical analysis was performed using two-tailed unpaired t-test; n=3; mean ±SD.

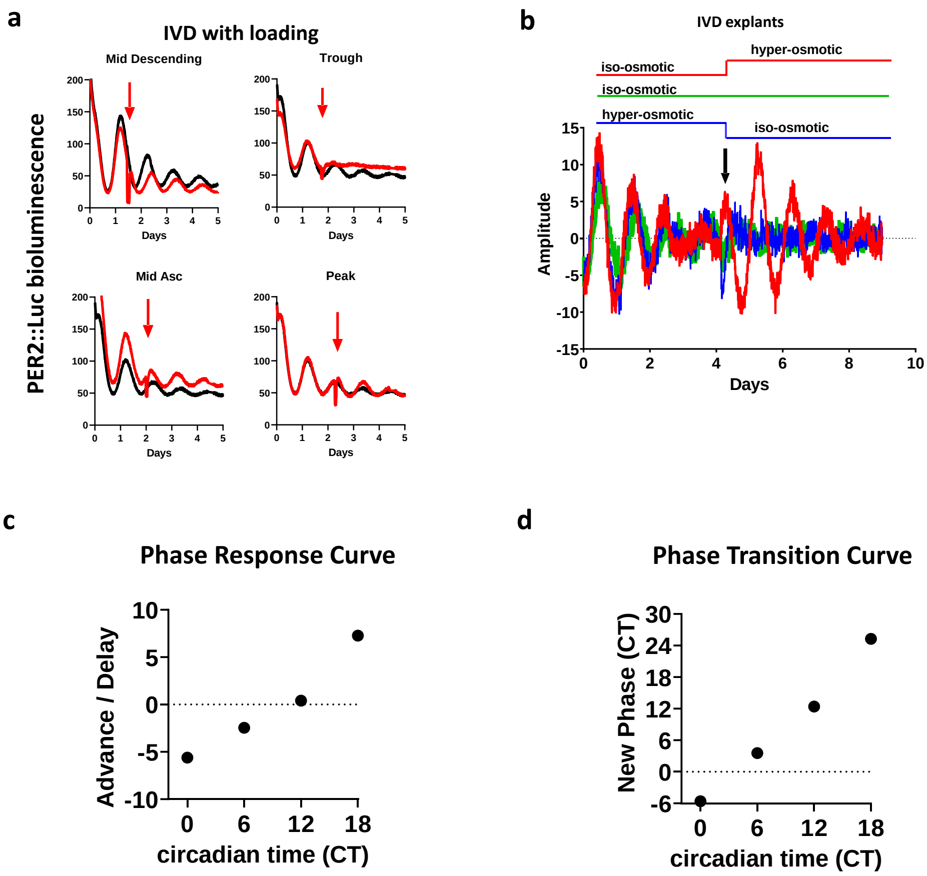

Figure S2. Hyperosmolarity has phase dependent synchronising effect on cartilage and IVD explants. (**a**) Bioluminescence recording of PER2::Luc mouse IVD explants. Starting at 36 hr (middle of descending phase of PER2::Luc) explants were subjected to mechanical loading (red arrow) at 6 hr intervals. Each trace represents the mean of 3 explants. (**b**) Bioluminescence recording of PER2::Luc mouse IVD explants cultured in iso- (330 mOsm) and hyper- (530 mOsm) osmotic conditions. At day 4, conditioned media were switched between iso-osmotic (red trace) and hyper-osmotic (blue trace) adapted explants. Control (black trace) was left undisturbed. Quantification of the phase response (**c**) and phase transition (**d**) after exposure of IVDs to +200 mOsm increase in osmolarity (corresponding to main Figure 2F).

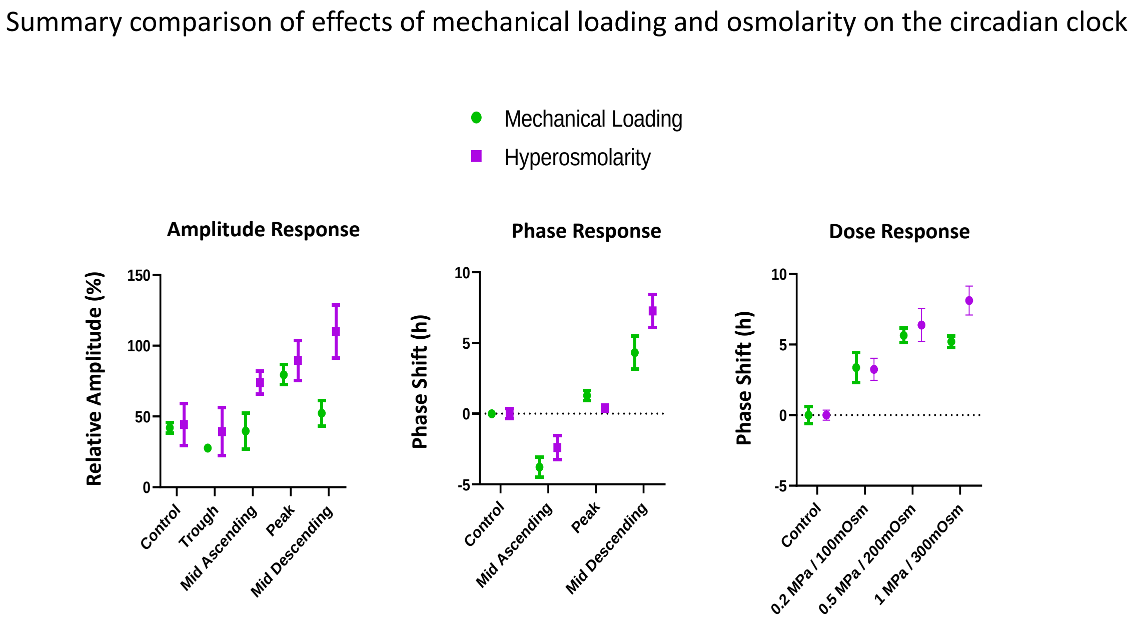

Figure S3. A summary comparison of the effects of mechanical loading and osmolarity on the circadian clock of cartilage tissue (mean ±SD).

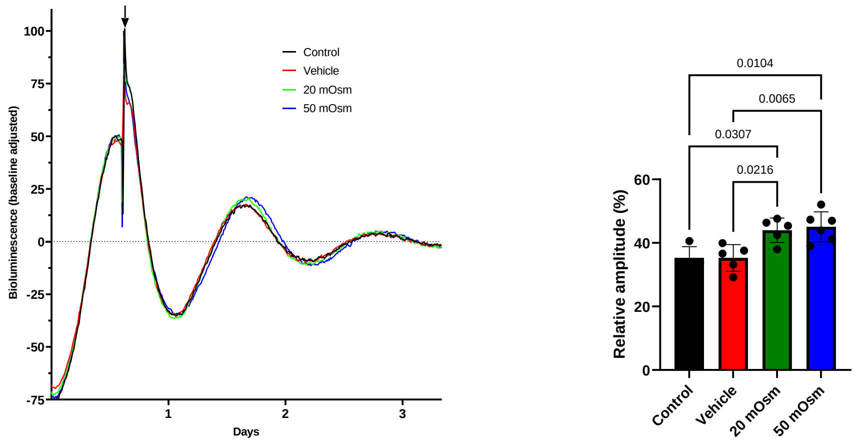

Figure S4. Bioluminescence recording of primary PER2::Luc mouse chondrocytes exposed to low level hyper-osmotic change. Traces represent mean of 5 cultures. At the peak of oscillation (black arrow), cells were exposed to +20 or +50 mOsm increase in osmolarity. Quantification of amplitude was carried out by calculating the peak after treatment as a % of the peak before treatment. N=5, One-way ANOVA, Tukeys multiple comparisons; mean ±SD. Source data are provided as a Source Data file.

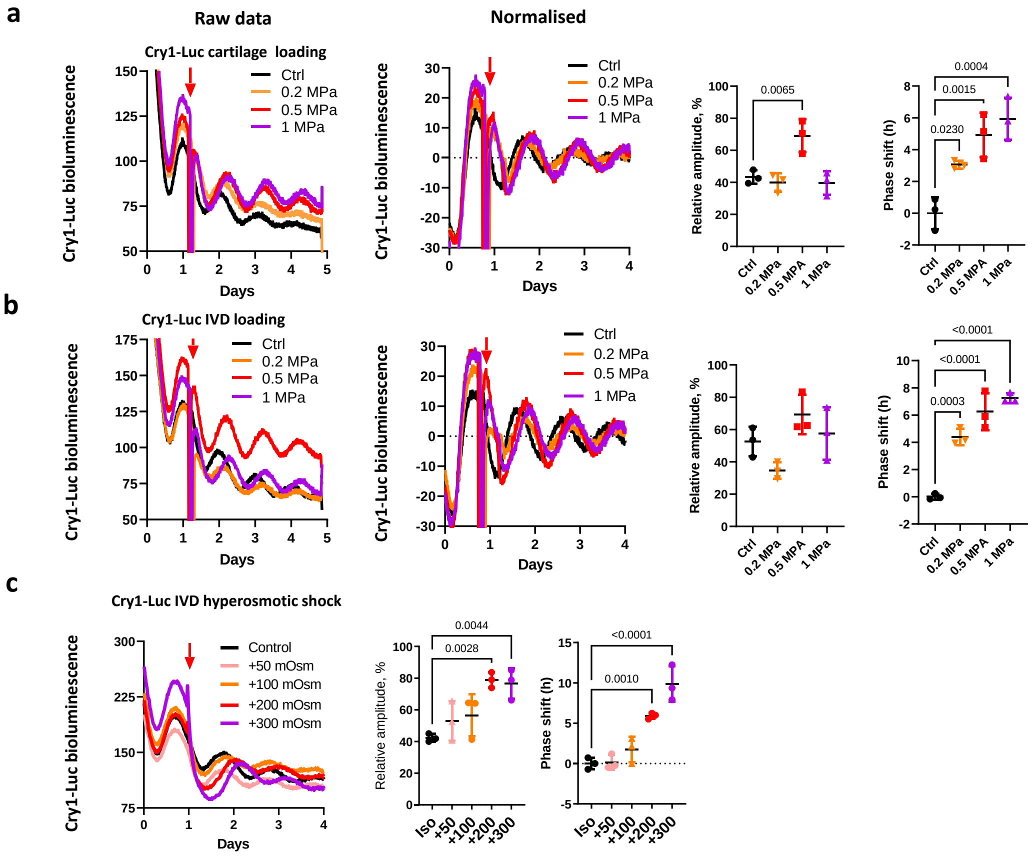

Figure S5. Hyperosmolarity and mechanical loading show dose dependent synchronising effect on cartilage and IVD explants of *Cry1*-Luc reporter mice. Bioluminescence recordings of *Cry1*-Luc reporter mouse femoral head cartilage (**a**) and IVD (**b**) explants. Raw bioluminescence counts (left) and normalised (right) were displayed. At ~30 hr of recording (red arrow) explants were subjected to mechanical loading (1 Hz, 1 hr) at compression magnitudes of 0.2 MPa (orange), 0.5MPa (red) and 1MPa (purple). Each trace represents the mean of 3 explants. Quantification (right) of amplitude and phase shift after mechanical loading (mean ±SD). (**c**) Bioluminescence recording and quantification of IVD explants from *Cry1*-Luc reporter mouse exposed to increasing hyperosmolarity (red arrow). Each trace represents the mean of 3 explants. Quantification of amplitude and phase shift after hyperosmolarity was displayed as mean ±SD. Statistical analysis was performed using one-way ANOVA. P values were adjusted for multiple comparisons using Dunnett's multiple comparisons test; mean ±SD. Source data are provided as a Source Data file.

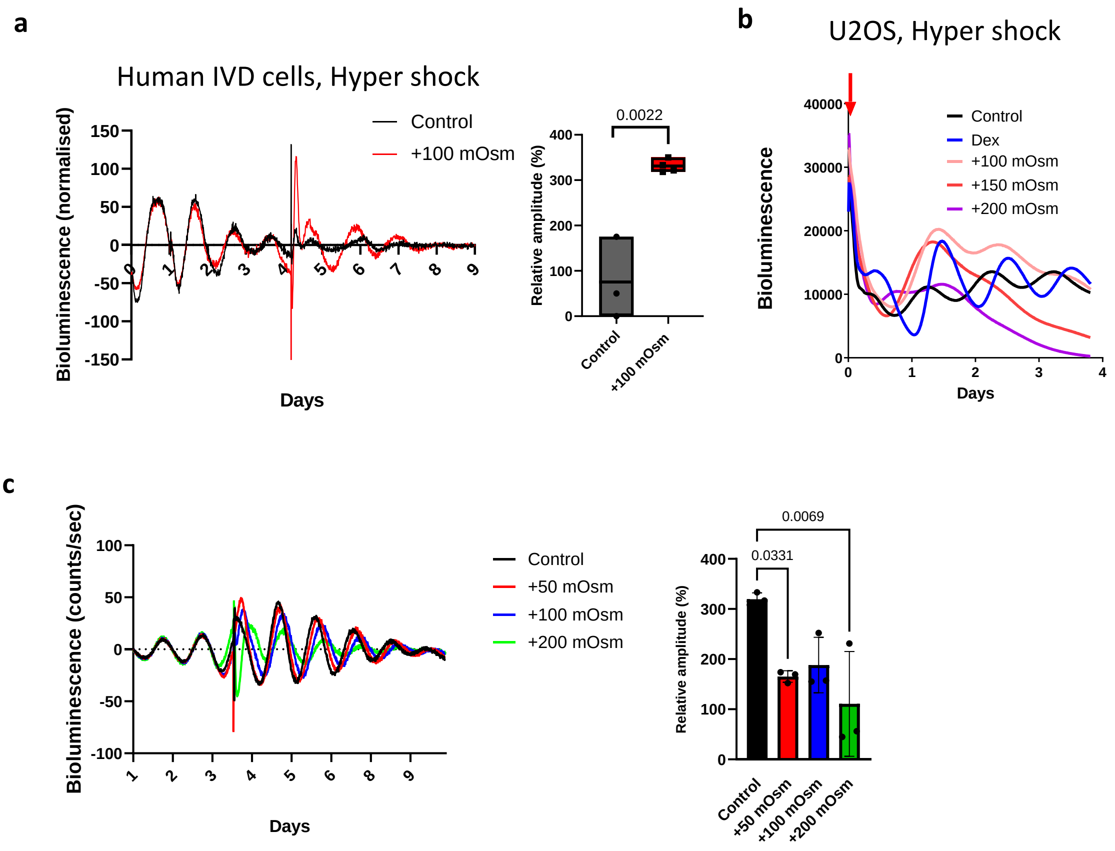

Figure S6. Hyperosmolarity has synchronising effect on human IVD cells but not on U2OS or HaCaT cells. (**a**) Representative bioluminescence recording of a human IVD cell line NP115 carrying *Per2*-Luc reporter exposed to +100 mOsm osmolarity increase (red arrow). n = 4. (**b**) Representative bioluminescence recording of U2OS cell line carrying *Per2*-Luc reporter. At the beginning of the experiment (red arrow) the cells were synchronised using dexamethasone (blue) or exposed to an increase in osmolarity. Black: unsynchronised, untreated control. n = 3. (**c**) Bioluminescence recording of HaCaT keratinocytes carrying *Per2*-Luc reporter. Each trace is a mean of 3 cultures. At the beginning of the experiment the cells were synchronised using dexamethasone and at day 3.5 exposed to increased osmolarity, control was untreated. Amplitude after treatments was quantified relative to amplitude before treatments. Statistical analysis was performed using student two-tailed unpaired t-test (a) and one-way ANOVA (c). P values were adjusted for multiple comparisons using Dunnett's multiple comparisons test; mean ±SD. Source data are provided as a Source Data file.

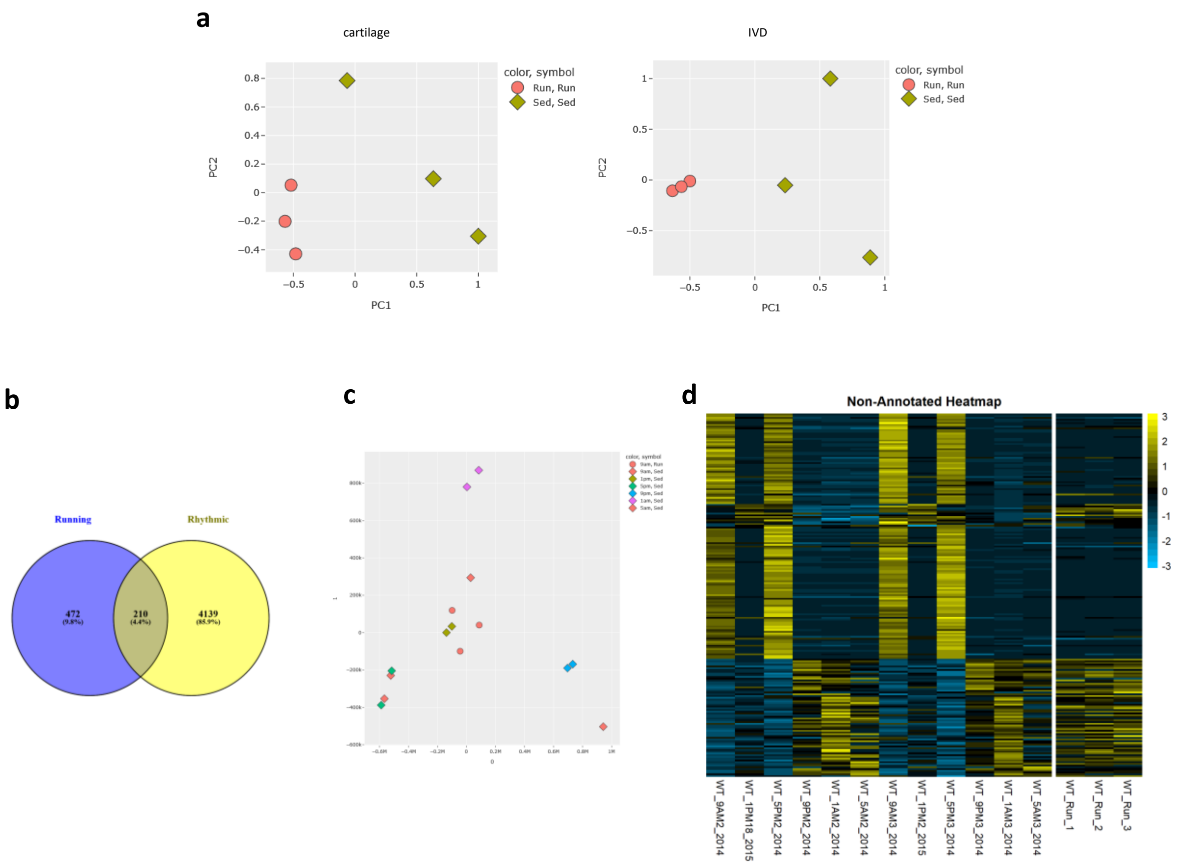

Figure S7. Overlap between rhythmic genes and genes regulated by treadmill running. (**a**) PCA plots of RNAseq data from cartilage and IVD samples of mice on treadmill running or sedentary. (**b**) Overlap between rhythmic genes and genes differentially regulated by running. (**c**) PCA plot comparing samples from treadmill running mice with samples from circadian time series (all genes in the datasets as opposed to rhythmic genes in main figure 4j). (**d**) Heatmap showing expression of the 210 overlapping genes from b.

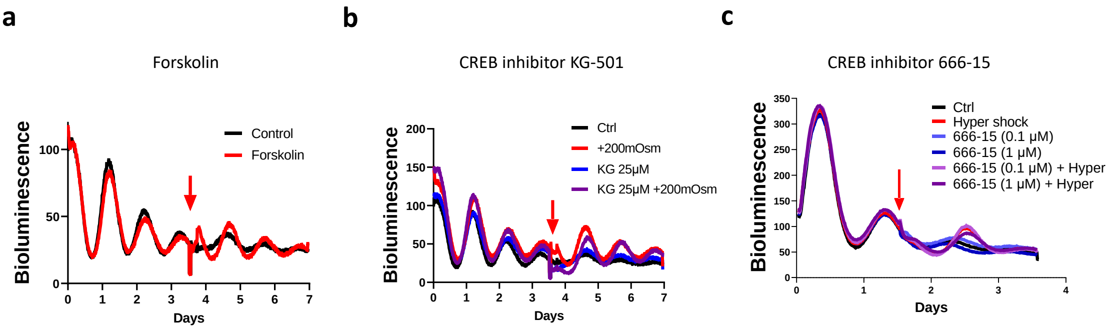

Figure S8. CREB pathway is not involved in the synchronising effect of hyperosmolarity. Bioluminescence recording of PER2::Luc mouse IVD explants. (**a**) At mid descending phase of day 3 explants were treated with forskolin (red arrow). (**b**) explants were exposed to +200 mOsm increase in osmolarity with or without a CREB inhibitor KG-501. (**c**) At 36 hr (red arrow) explants were exposed to +200 mOsm increase in osmolarity with or without a CREB inhibitor 666-15. Controls: no treatment, inhibitor only. Each trace represents the mean of 3 explants.

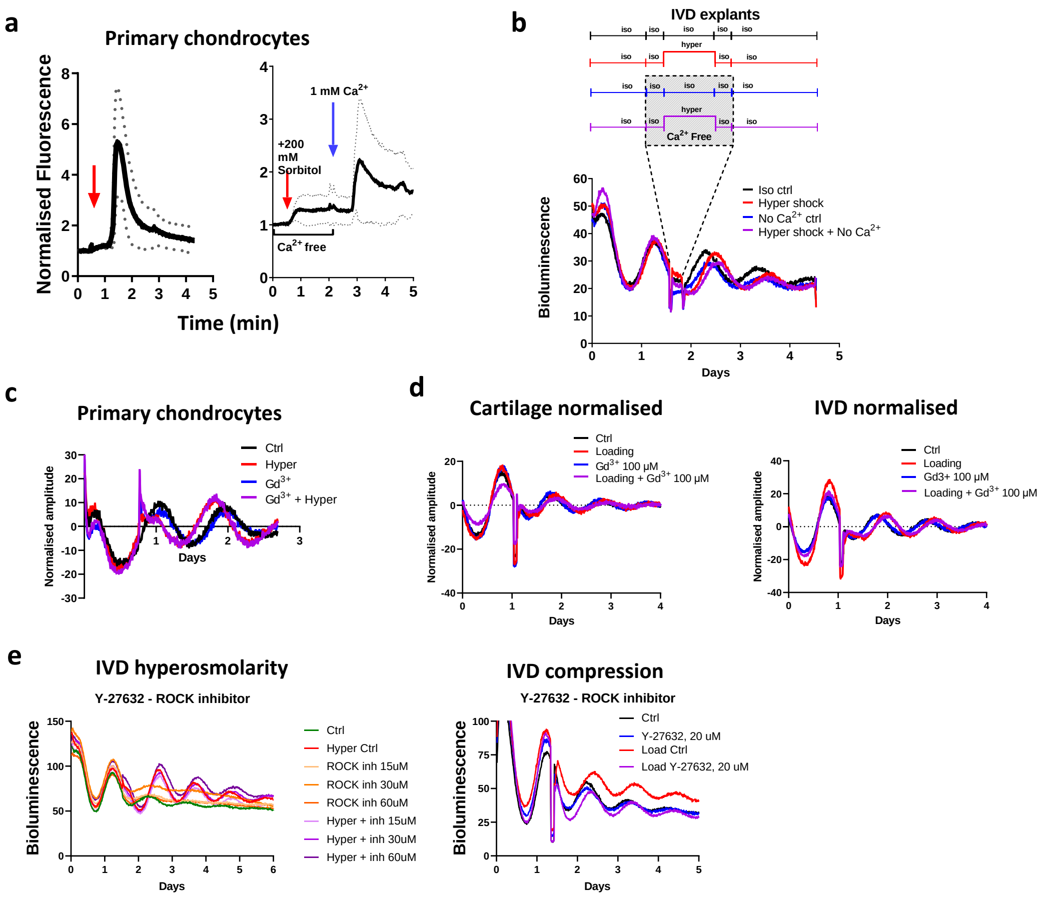

Figure S9. Plasma membrane calcium channels or the ROCK pathway are not involved in the synchronising effect of hyperosmolarity and mechanical loading. (**a**) Calcium imaging in mouse primary chondrocytes using Fluo-4. Reaction of chondrocytes to +200 mOsm increase in osmolarity (red arrow) in calcium-containing medium (left) and in calcium-free medium (right). Blue arrow indicates re-introduction of calcium to the medium. Mean ±SD of fluorescence signals from 15 cells. (**b**) Bioluminescence recordings of PER2::Luc reporter mouse IVD explants. At mid descending phase the explants were pre-incubated in iso-osmotic calcium-free medium (blue and purple traces). Explants were exposed to +200 mOsm increase in osmolarity for 6 hr (red and purple) and returned to iso-osmotic for 1 hr post-incubation (blue and purple traces calcium-free). Subsequently media for all explants was changed to calcium-containing iso-osmotic medium. Black trace: calcium-containing iso-osmotic control. Blue trace: calcium-free incubation iso-osmotic control. Each trace represents the mean of 4 explants. (**c**) Bioluminescence recording of PER2::Luc mouse primary chondrocytes exposed at mid ascending phase to +200 mOsm increase in osmolarity with or without a calcium channel blocker gadolinium (Gd3+). (**d**) Bioluminescence recording of PER2::Luc mouse femoral head cartilage (left) and IVDs. At mid descending phase explants were exposed to mechanical loading (1 Hz, 1 hr, 0.5MPa) with or without a calcium channel blocker gadolinium (purple and red). No loading gadolinium control was shown in blue and no treatment control in black. Each trace represents the mean of 3 explants. (**e**) Bioluminescence recording of PER2::Luc mouse IVDs. At mid descending phase explants were exposed to mechanical loading +200 mOsm increase in osmolarity with or without a ROCK inhibitor Y-27632. Each trace represents the mean of 3 explants.

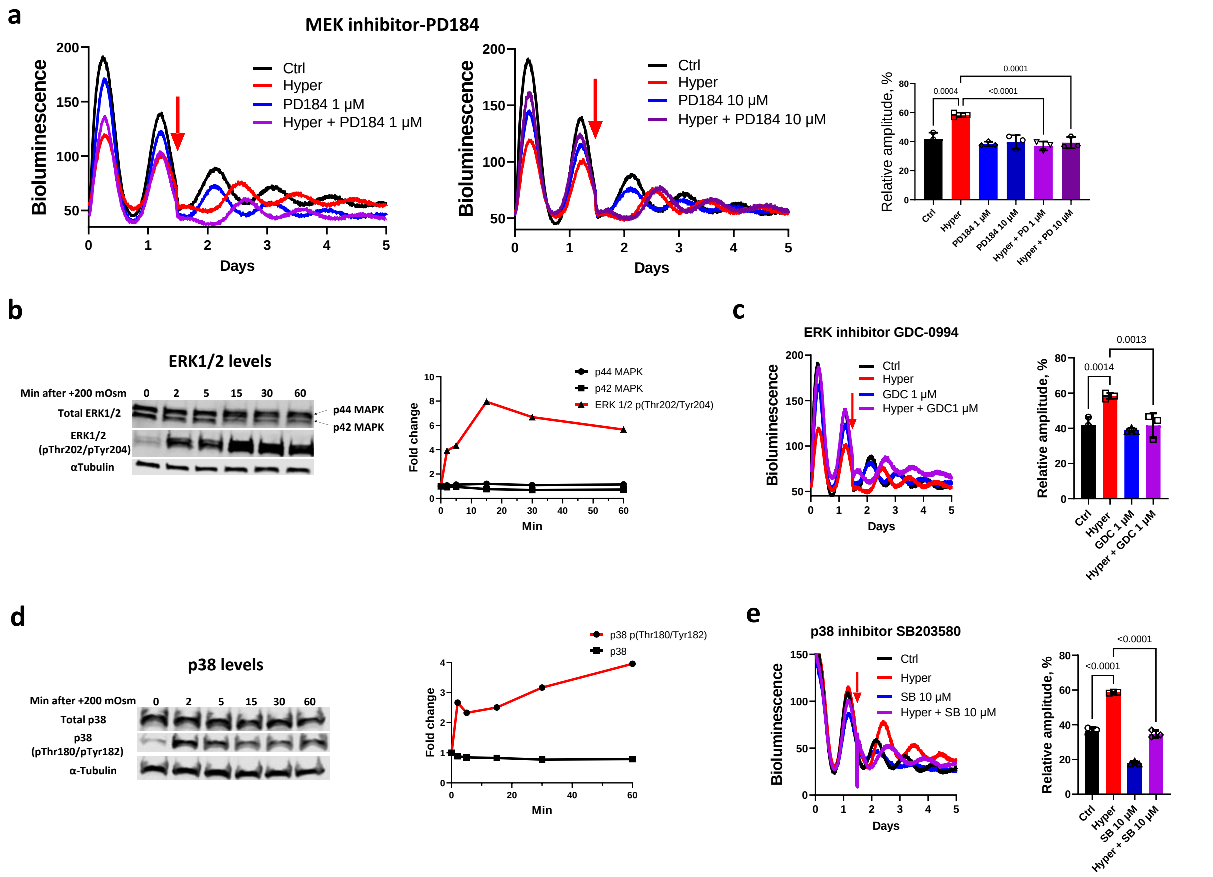

Figure S10. Inhibition of the MEK/ERK pathway or the p38 kinase blocks the synchronising effect of hyperosmolarity. (**a**) Bioluminescence recording of PER2::Luc mouse IVD explants. At mid descending phase explants were exposed to +200 mOsm increase in osmolarity (red arrow) with or without a MEK inhibitor PD184352 (purple and red trace). Blue: inhibitor control. Black: no treatment control. Each trace represents the mean of 3 explants. Amplitude after hyperosmotic challenge was quantified as % of the amplitude of the peak before challenge. Mean ±SD. (**b**) WB and quantification (relative to αTubulin) showing phosphorylation of ERK1/2 at Thr202/Tyr204 in mouse primary chondrocytes following +200 mOsm change in osmolarity. (**c**) Bioluminescence recording and amplitude quantification of PER2::Luc mouse IVD explants. At 3 6hr (mid descending phase, red arrow) explants were exposed to +200 mOsm increase in osmolarity with or without the ERK1/2 inhibitor GDC-0994. Controls: no treatment, inhibitor only. (**d**) WB and quantification (relative to αTubulin) showing phosphorylation of p38 at Thr180/Tyr182 in mouse primary chondrocytes following +200 mOsm change in osmolarity. (**e**) Bioluminescence recording and amplitude quantification of PER2::Luc mouse IVD explants. At 36 hr (mid descending phase, red arrow) explants were exposed to +200 mOsm increase in osmolarity with or without the p38 inhibitor SB203580. Controls: no treatment, inhibitor only. Amplitude of the peak after treatment was expressed as

% of the amplitude of the peak before treatment. Mean ±SD. Statistical analysis was performed using one-way ANOVA. P values were adjusted for multiple comparisons using Dunnett's multiple comparisons test. Bars mean ±SD. Source data are provided as a Source Data file.

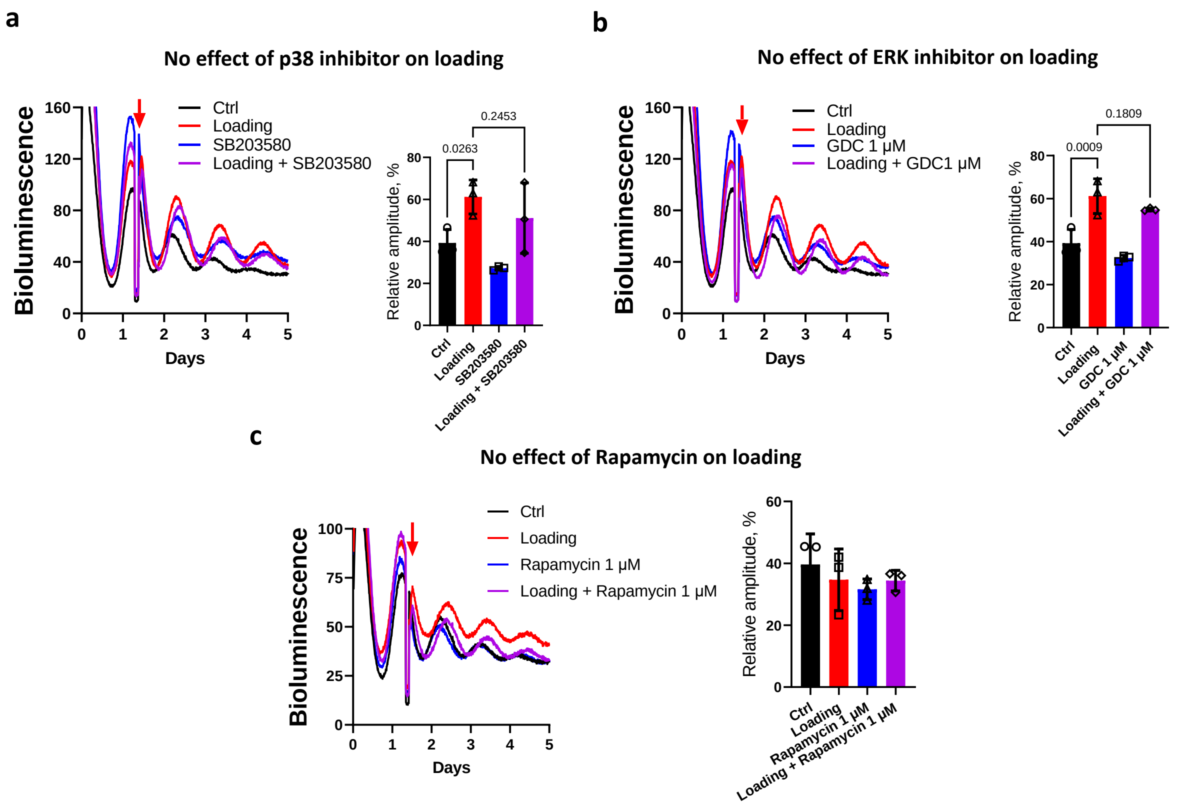

Figure S11. The MEK/ERK pathway, p38 kinase or mTORC1 are not involved in the synchronising effect of mechanical loading. Bioluminescence recording and amplitude quantification of PER2::Luc mouse femoral head cartilage explants. At 36 hr (approximately peak of PER2) explants were exposed to mechanical loading (0.5 MPa, 1 Hz, 1 hr) with or without the p38 inhibitor SB203580 (**a**), the ERK1/2 inhibitor GDC-0994 (**b**) and the mTORC1 inhibitor rapamycin (**c**). Each trace represents the mean of 3 explants. Amplitude after loading was quantified as % of the amplitude of the peak before loading. Mean ±SD. Statistical analysis was performed using one-way ANOVA. P values were adjusted for multiple comparisons using Dunnett's multiple comparisons test. Source data are provided as a Source Data file.

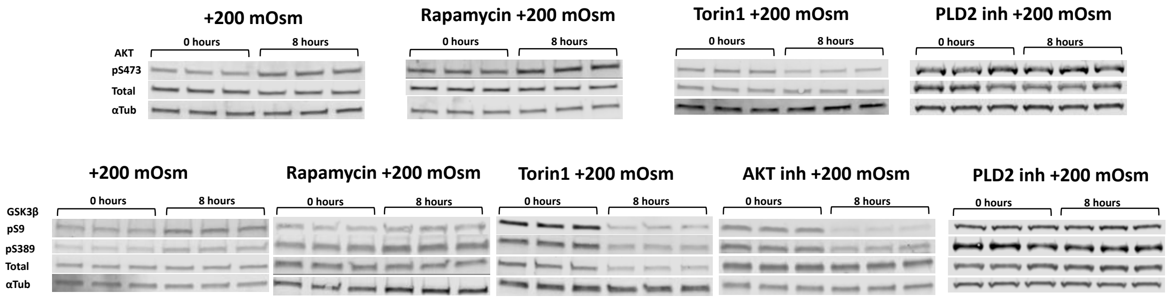

Figure S12. Western blot images quantified in main Figure 5. Mouse primary chondrocytes were exposed to +200 mOsm increase in osmolarity with or without various inhibitors and harvested 8 hours later.
