# Supplementary material for: Mechanical loading and hyperosmolarity as a daily resetting cue for skeletal circadian clocks
Source: Nat Commun. 2023 Nov 14;14:7237. doi: 10.1038/s41467-023-42056-1 (PMC10646113; doi:10.1038/s41467-023-42056-1)
Supplement: Supplementary file 3 — Description of Additional Supplementary Files [file 41467_2023_42056_MOESM3_ESM.pdf]

Description of Additional Supplementary Files

Supplementary Data 1: Primary Chondrocytes Osmotic stress RNAseq Rhythmic Genes

Supplementary Data 2: Primary Chondrocytes Osmotic stress RNAseq T0 vs T4 DEGs

Supplementary Data 3: Primary Chondrocytes Osmotic stress RNAseq Rhythmic Canonical Pathways

Supplementary Data 4: Primary Chondrocytes Osmotic stress RNAseq Rhythmic Upstream Regulators

Supplementary Data 5: Primary Chondrocytes Osmotic stress RNAseq T0 vs T4

Supplementary Data 6: Primary Chondrocytes Osmotic stress RNAseq T0 vs T4

Supplementary Data 7: Cartilage Running WT DEGs

Supplementary Data 8: IVD Running WT DEGs

Supplementary Data 9: Cartilage Running Canonical Pathways

Supplementary Data 10: Cartilage Running Upstream Regulators

Supplementary Data 11: IVD Running Canonical Pathways

Supplementary Data 12: IVD Running Upstream Regulators

Supplementary Movie 1: Bioluminescence imaging of PER2::Luc mouse spine segment. The tissue was synchronised with Dex at the beginning of recording. After 3 days sorbitol was added to increase osmolarity by +200 mOsm.
