# Supplementary material for: Mechanical loading and hyperosmolarity as a daily resetting cue for skeletal circadian clocks
Source: Nat Commun. 2023 Nov 14;14:7237. doi: 10.1038/s41467-023-42056-1 (PMC10646113; doi:10.1038/s41467-023-42056-1)
Supplement: Supplementary file 6 — Reporting Summary [file 41467_2023_42056_MOESM6_ESM.pdf]

nature portfolio

Corresponding author(s): Qing-Jun Meng and Judith Hoyland

Last updated by author(s): Aug 24, 2023

Reporting Summary

Nature Portfolio wishes to improve the reproducibility of the work that we publish. This form provides structure for consistency and transparency in reporting. For further information on Nature Portfolio policies, see our Editorial Policies and the Editorial Policy Checklist.

Statistics

For all statistical analyses, confirm that the following items are present in the figure legend, table legend, main text, or Methods section.

n/a	Confirmed	
[ ]	[x]	The exact sample size (*n*) for each experimental group/condition, given as a discrete number and unit of measurement
[ ]	[x]	A statement on whether measurements were taken from distinct samples or whether the same sample was measured repeatedly
[ ]	[x]	The statistical test(s) used AND whether they are one- or two-sided<br>*Only common tests should be described solely by name; describe more complex techniques in the Methods section.*
[x]	[ ]	A description of all covariates tested
[ ]	[x]	A description of any assumptions or corrections, such as tests of normality and adjustment for multiple comparisons
[ ]	[x]	A full description of the statistical parameters including central tendency (e.g. means) or other basic estimates (e.g. regression coefficient) AND variation (e.g. standard deviation) or associated estimates of uncertainty (e.g. confidence intervals)
[ ]	[x]	For null hypothesis testing, the test statistic (e.g. *F*, *t*, *r*) with confidence intervals, effect sizes, degrees of freedom and *P* value noted<br>*Give P values as exact values whenever suitable.*
[x]	[ ]	For Bayesian analysis, information on the choice of priors and Markov chain Monte Carlo settings
[x]	[ ]	For hierarchical and complex designs, identification of the appropriate level for tests and full reporting of outcomes
[x]	[ ]	Estimates of effect sizes (e.g. Cohen's *d*, Pearson's *r*), indicating how they were calculated

Our web collection on statistics for biologists contains articles on many of the points above.

Software and code

Policy information about availability of computer code

Data collection	LumiCycle (Actimetrics) - bioluminescence recording; Zeiss software Aim version 4.2 -calcium imaging; Zeiss Zen2 software - Venus reporter fluorescence; Image Studio (Li-Cor)- fluorescent western blot imaging
Data analysis	LumiCycle Analysis 2.31 (Actimetrics) - bioluminescence; GraphPad Prism 9 - basic statistical analysis; Cell Tracker (version 0.6) - Venus reporter fluorescence analysis; RNAseq analysis: FastQC 0.11.9, Trimmomatic_0.39, STAR_2.7.7a, DESeq2_1.28.1, scikit-learn 1.1.0, MetaCycle_1.2.0, Ingenuity Pathway Analysis (Qiagen), Image Studio Lite 5.2 (Li-Cor)- fluorescent western blot imaging

For manuscripts utilizing custom algorithms or software that are central to the research but not yet described in published literature, software must be made available to editors and reviewers. We strongly encourage code deposition in a community repository (e.g. GitHub). See the Nature Portfolio guidelines for submitting code & software for further information.

Data

Policy information about availability of data

All manuscripts must include a data availability statement. This statement should provide the following information, where applicable:

- Accession codes, unique identifiers, or web links for publicly available datasets
- A description of any restrictions on data availability
- For clinical datasets or third party data, please ensure that the statement adheres to our policy

RNAseq raw data were deposited to ArrayExpress repository (accession no. E-MTAB-11040 for osmotic stress in primary chondrocytes and E-MTAB-12877 for treadmill running tissues). Circadian cartilage RNAseq dataset E-MTAB-3428 was used for comparison with the treadmill running dataset. Mouse reference genome

mm10/GRCm38 was used for mapping RNAseq reads. All other data is contained within the manuscript and supplementary information.

Human research participants

Policy information about studies involving human research participants and Sex and Gender in Research.

Reporting on sex and gender	N/A
Population characteristics	N/A
Recruitment	N/A
Ethics oversight	N/A

Note that full information on the approval of the study protocol must also be provided in the manuscript.

Field-specific reporting

Please select the one below that is the best fit for your research. If you are not sure, read the appropriate sections before making your selection.

[x] Life sciences   [ ] Behavioural & social sciences   [ ] Ecological, evolutionary & environmental sciences

For a reference copy of the document with all sections, see nature.com/documents/nr-reporting-summary-flat.pdf

Life sciences study design

All studies must disclose on these points even when the disclosure is negative.

Sample size	Sample sizes were based on similar experiments done with similar cell lines and explants (https://doi.org/10.1172/JCI82755, http://dx.doi.org/10.1136/annrheumdis-2016-209428 and https://doi.org/10.1016/j.matbio.2023.07.002). We did not perform power estimates prior to collecting the data and performed statistical tests after data collection.
Data exclusions	No data was excluded from analysis.
Replication	6 mice per condition were used in treadmill running experiments. Between 3-6 mice per condition were used in in tissue explant experiments as indicated in figure legends. All experiments were successfully replicated and results showed consistency.
Randomization	Samples and animals were randomly assigned to control and treatment conditions.
Blinding	Investigators were not blinded during data collection and analysis. Experimental conditions (e.g. young vs. old mice or running vs. sedentary) and data analysis methods did not allow for blinding.

Reporting for specific materials, systems and methods

We require information from authors about some types of materials, experimental systems and methods used in many studies. Here, indicate whether each material, system or method listed is relevant to your study. If you are not sure if a list item applies to your research, read the appropriate section before selecting a response.

Materials & experimental systems

n/a	Involved in the study	
[ ]	[x]	Antibodies
[ ]	[x]	Eukaryotic cell lines
[x]	[ ]	Palaeontology and archaeology
[ ]	[x]	Animals and other organisms
[x]	[ ]	Clinical data
[x]	[ ]	Dual use research of concern

Methods

n/a	Involved in the study	
[x]	[ ]	ChIP-seq
[x]	[ ]	Flow cytometry
[x]	[ ]	MRI-based neuroimaging

Antibodies

Antibodies used	All primary antibodies were purchased from Cell Signaling Technology ERK1/2 (#4695), pThr202/pTyr204 ERK1/2 (#4370), p38 (#8690), pThr180/pTyr182 p38 (#4511), GSK3β (#12456), pSer9 GSK3β (#5558), except for pSer389 GSK3β (14850-1-AP) which was purchased from Proteintech and αTubulin (T9026) purchased from Sigma Aldrich. Secondary antibodies: LI-COR

	IRDye® 800CW Goat anti-Mouse IgG (926-32210) and IRDye® 680RD Goat anti-Rabbit IgG (926-68071).
Validation	Antibodies were validated by manufacturer by either knock-down of target, overexpression of target or treatments inducing expression/phosphorylation of target. All antibodies were validated by manufacturer for the species (Human, Mouse) and application (western blotting) used in this study. Detected protein size was checked against uniprot database and manufacturers information sheet.

Eukaryotic cell lines

Policy information about cell lines and Sex and Gender in Research

Cell line source(s)	Primary chondrocytes were isolated from PER2::Luc reporter mice (Yoo, S.H., et al. PNAS 2013) and PER2::Venus reporter mice (Smyllie, N.J, et al. Curr Bio 2016). Immortalised human nucleus pulposus (NP) cell line NP115 was described previously (van den Akker, G.G., et al. Arthritis Res Ther, 2014). Immortalized rat NP cells was described previously (Oh CD, et al. Spine (Phila Pa 1976) 2016).
Authentication	Primary chondrocytes were isolated and phenotyped according to published protocol (Gosset M, et al. Nat Protoc 2008). Particularly at confluence primary mouse chondrocytes exhibit the typical chondrocyte morphology, with a rounded or polygonal shape and granular cytoplasm. Expression of chondrocyte markers Col2a1 and Acan was confirmed by qPCR using gene and species specific TaqMan probes.
Mycoplasma contamination	Cells are routinely tested. No contamination was found.
Commonly misidentified lines (See ICLAC register)	No commonly misidentified cell lines were used in this study.

Animals and other research organisms

Policy information about studies involving animals; ARRIVE guidelines recommended for reporting animal research, and Sex and Gender in Research

Laboratory animals	PER2::Luc reporter mice (Yoo, S.H., et al. PNAS 2013) and PER2::Venus reporter mice (Smyllie, N.J, et al. Curr Bio 2016) were used at 4-8 weeks of age. Additionally PER2::Luc reporter mice at 12 months old were used for ageing experiment. Mice were housed in Tecniplast Green Line cages and racks at 20-22°C and average 60% humidity, with standard rodent chow available ad libitum and under 12:12 hr light dark schedule (light on at 7 am; light off at 7 pm).
Wild animals	No wild animals were used.
Reporting on sex	Mice of both sexes were used in experiments
Field-collected samples	none
Ethics oversight	All animal studies were performed in accordance with the 1986 UK Home Office Animal Procedures Act. Approval was provided by the University o Manchester's Animal Welfare and Ethical Review Board (AWERB).

Note that full information on the approval of the study protocol must also be provided in the manuscript.
